# Influence of Tumor-Treating Fields in a Young Patient With Primary Spinal Glioblastoma Multiforme (GBM): A Case Report of a Rare Tumor

**DOI:** 10.7759/cureus.49441

**Published:** 2023-11-26

**Authors:** Emmanuel K Mikobi, Gerard Voorhees, Aftab Mahmood, Arpit Gandhi, Michael Bailey, Jacqueline Phillips

**Affiliations:** 1 Internal Medicine, Corpus Christi Medical Center, Corpus Christi, USA; 2 Radiation Oncology, Corpus Christi Medical Center, Corpus Christi, USA; 3 Hematology and Oncology, Corpus Christi Medical Center, Corpus Christi, USA; 4 Radiology, Radiology Associates, Corpus Christi, USA; 5 Pathology, Corpus Christi Medical Center, Corpus Christi, USA; 6 Internal Medicine, Graduate Medical Education, Corpus Christi Medical Center, Corpus Christi, USA

**Keywords:** ttfields, tumor-treating fields, spinal gbm, primary thoracic spine gbm, alternating electrical field therapy, primary spinal glioblastoma multiforme

## Abstract

We present a 31-year-old female patient with primary glioblastoma multiforme (GBM) of the thoracic spine, diagnosed in approximately mid-2020. Her symptoms began several months prior with right foot paresthesia, which progressed to neuropathy ascending from her distal to proximal right lower extremity. Over several months, she developed lumbo-thoracic throbbing pain, which was dermatomal radiating anteriorly. Her pain worsened with activity. A thoracic spine MRI showed a focus of abnormal intradural intramedullary enhancement present from the T10-T11 disc level to the T12-L1 disc level, producing a large amount of edema within the cord. She underwent a gross total surgical resection. The patient had WHO Grade IV spinal GBM per histopathology. The patient received adjuvant concurrent radiation therapy and temozolomide chemotherapy. She continues with maintenance temozolomide along with the compassionate use of Novocure alternating electrical field therapy for the spine. She is being monitored closely by a multi-specialty team. At 32 months post-radiation therapy, her disease is stable with no evidence of progression. She has made significant improvements in her ambulation and symptoms.

While GBM is most commonly intracranial, primary spinal GBM is relatively rare. Although established treatment guidelines exist for supratentorial GBM, treatment protocol choices for spinal GBM remain controversial but typically mirror those used for intracranial GBM and include surgery, radiation therapy, and chemotherapy. Alternating electrical field therapy, also known as tumor-treating fields (TTFields), is indicated for adjuvant treatment of intracranial GBM. While further studies of TTFields in spinal GBM are needed, TTFields appear to be a safe adjunct treatment for spinal GBM. Further studies are still needed aimed at finding an improved treatment for spinal GBM.

## Introduction

We present the case of a 31-year-old female patient with the diagnosis of primary spinal glioblastoma multiforme (GBM). This patient underwent resection of her spinal intramedullary tumor, followed by postoperative radiation therapy with concurrent temozolomide. She has been maintained on temozolomide along with tumor-treating field (TTFields) therapy for 32 months. Tumor-treating fields are alternating electric fields that continuously and selectively disrupt cancer cell division in solid tumors by an anti-microtubule mechanism of action [1]. Apart from surgery, radiation, and chemotherapy, alternating electrical field therapy is indicated in the treatment of supratentorial GBM per the National Comprehensive Cancer Network (NCCN) guidelines [2]. The use of TTFields in addition to maintenance temozolomide chemotherapy vs. temozolomide alone has been shown to improve disease-free survival in patients with GBM [3, 4].

## Case presentation

At the time of this publication, the patient is a 33-year-old female, nulligravida and nullipara (G0P0), with a history of endometriosis, generalized anxiety disorder, and depression. She was diagnosed with primary spinal cord GBM in approximately mid-2020. Her symptoms began towards the end of 2019 with a constant and unremitting right foot paresthesia. In early 2020, the paresthesia and subsequent neuropathy began to ascend her distal right lower extremity, finally involving her proximal right lower extremity several months later. Over the next few months, she developed lumbo-thoracic throbbing pain, which was dermatomal radiating anteriorly and worsened with activity.

**Figure 1:**
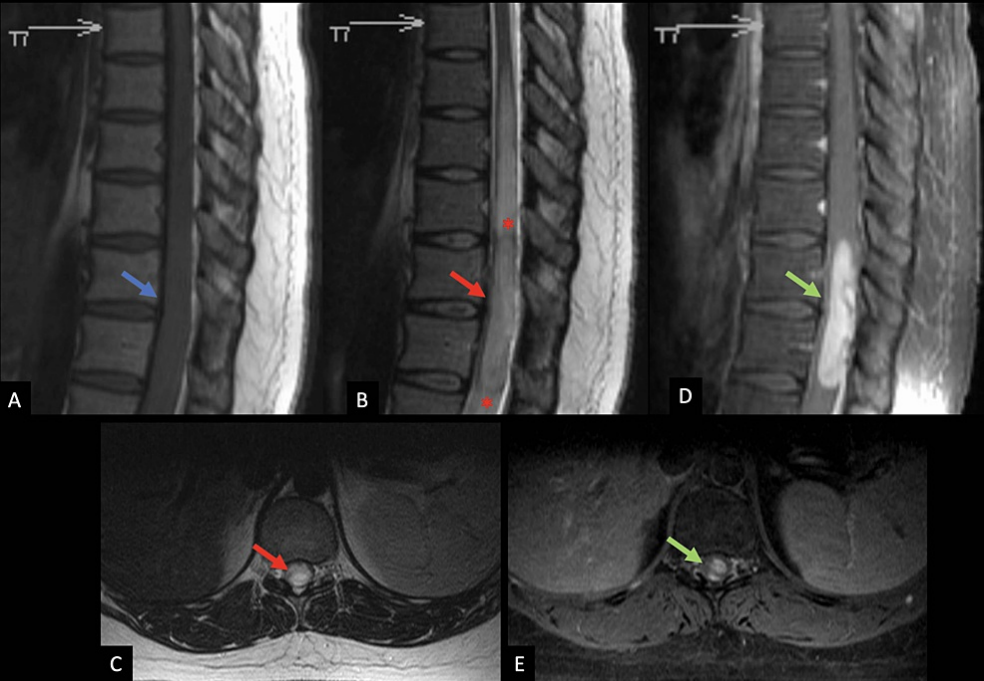
Preoperative MR imaging of the intramedullary mass in the lower thoracic cord An MRI of the thoracic spine from approximately mid-2020. Sagittal (A) the T1 weighted image (T1WI) shows an isointense signal mass in the lower thoracic cord at T11-T12 levels (blue arrow). The mass is more conspicuous and hyperintense in the signal on the sagittal (B) and axial (C) T2 weighted images (T2WIs) (red arrows). There is a subtle hypointense signal at its cranial and caudal margins (red asterisks). Extensive vasogenic edema is present from the T8 level to the conus (inferior extent not imaged). The mass demonstrates avid, mildly heterogenous contrast enhancement on sagittal (D) and axial (E) fat-suppressed, postcontrast T1WI (green arrows).

In approximately mid-2020, an MRI of the thoracic spine with and without contrast (Figure 1) showed a focus of abnormal intradural intramedullary enhancement present from the T10-T11 disc level to the T12-L1 disc level, producing a large amount of edema within the cord.

She was referred for a neurosurgical evaluation. Within several days following the initial MRI, she underwent T9, T10, and T11 thoracic laminectomies, exploration of an intradural intramedullary tumor, midline myelotomy, and resection of an intradural intramedullary tumor with standard microsurgical technique.

Pathology findings

**Figure 2:**
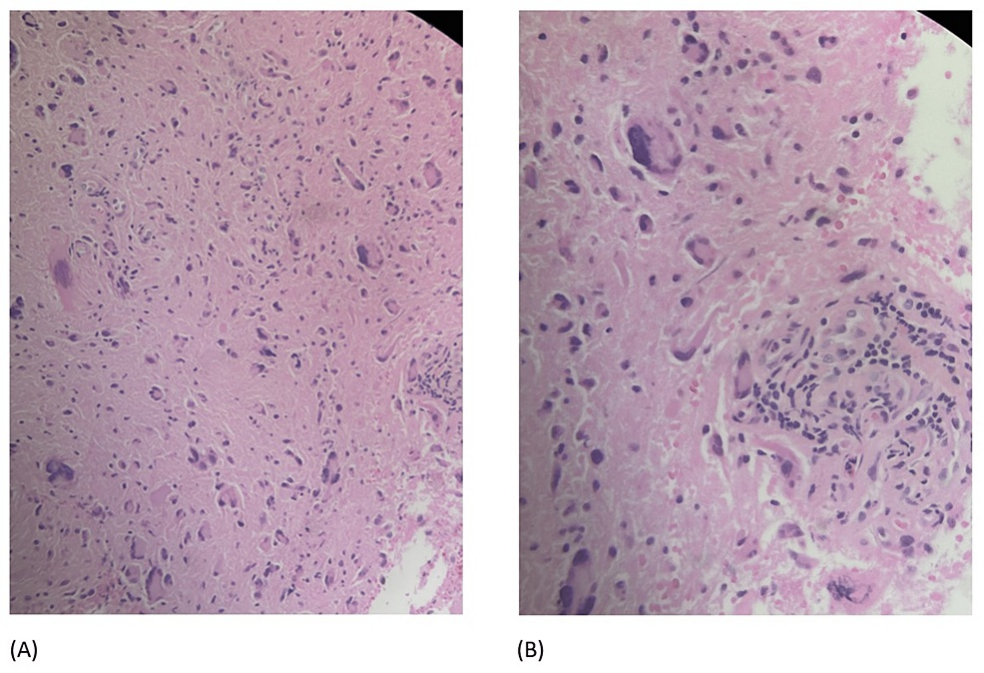
(A) Hypercellularity with large malignant astrocytes; (B) Hypercellularity with large malignant astrocytes and vascular proliferation

The histopathology report showed diffuse astrocytoma, giant cell glioblastoma, not otherwise specified (NOS), and WHO grade IV. Immunohistochemical stains for BRAF V600E, H3K27M, and H3K27me3 showed the tumor was negative for BRAF V600E and H3K27M, and staining for H3K27me3 was retained/normal. The H&E morphologic features and p53 immunopositivity were most consistent with the giant cell subtype of glioblastoma. The pathology slides are in Figure 2.

Treatment and follow-up

**Figure 3:**
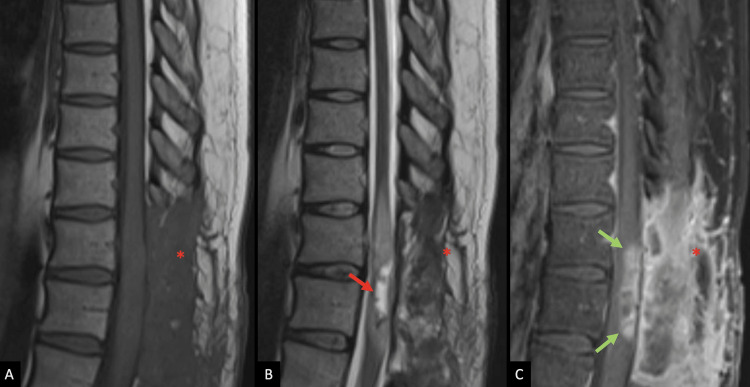
Postoperative MR imaging An MRI of the thoracic spine, approximately two months following the initial preoperative MRI in 2020. Sagittal (A) T1 weighted image (T1WI), (B) T2 weighted image (T2WI), and (C) fat-suppressed, post-contrast T1WI demonstrate changes following posterior decompression at the T10-L1 levels and subtotal resection of the intramedullary tumor. There is T2 hyperintense cystic change at the resection site (red arrow) with surrounding residual, enhancing tumor (green arrows). The extent of vasogenic edema has significantly decreased since the preoperative exam. There are expected post-surgical changes in the dorsal soft tissues (red asterisks).

**Figure 4:**
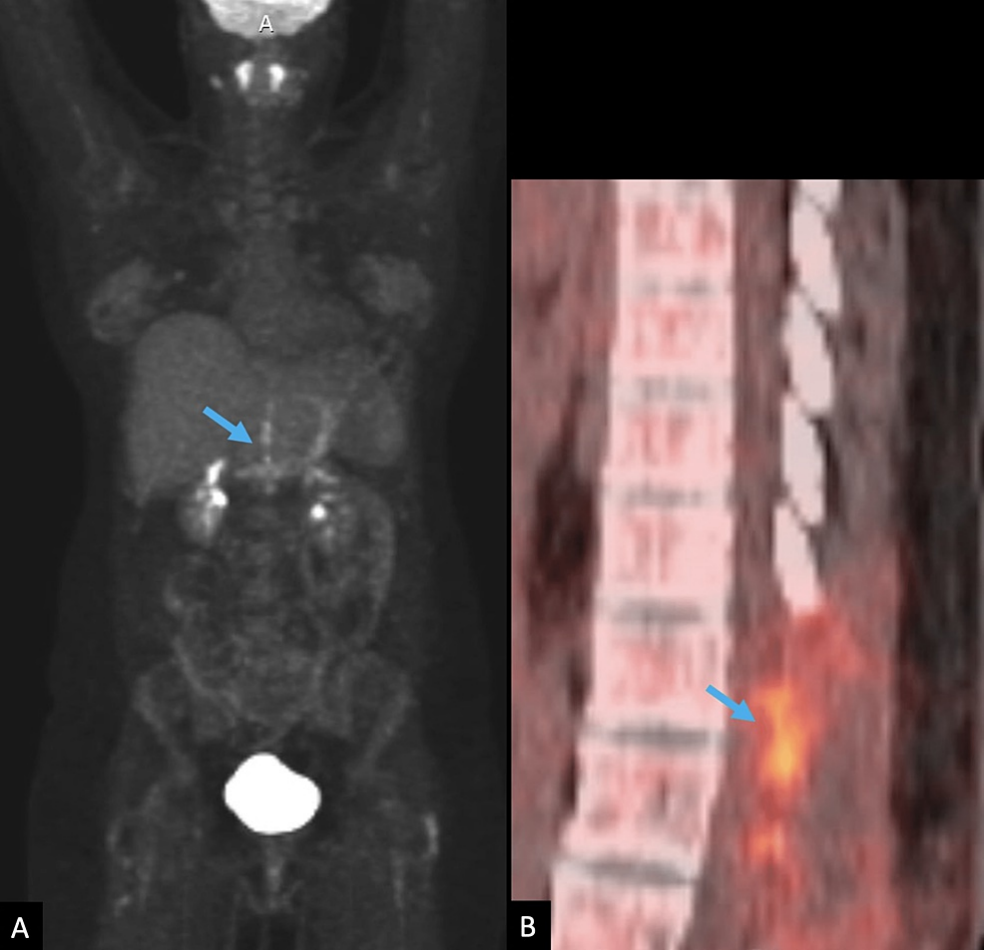
Postoperative PET/CT imaging A PET/CT, approximately two months following the initial preoperative MRI in 2020. Whole-body maximum intensity projection (MIP) PET (A) and sagittal (B) fused PET/CT show linearly increased F-fluorodeoxyglucose (FDG) uptake at the surgical site, representing residual tumor and postoperative change (blue arrows). There is no malignant FDG-avid disease elsewhere on the exam.

During rehabilitation, she was referred for medical oncology and radiation oncology evaluations. Postoperative thoracic MRI and PET scans were obtained, and they indicated residual tumors and postoperative changes (Figures 3-4).

She began radiation therapy approximately three months postoperatively. Over a five-week period, the patient received intensity-modulated, image-guided radiation therapy and volumetric arc therapy. A total tumor dose of 5000 cGy was delivered in 25 fractions (200 cGy/fx) to a field encompassing T10 through L1.

She received concurrent chemotherapy using temozolomide (75 mg/m^2^) 140 mg orally once a day. Following the completion of her concurrent radiation and chemotherapy, she began TTFields treatment using the Novocure Optune device. One month following chemoradiation, an MRI of her lumbar spine showed post-surgical changes of the lower thoracic spine laminectomies with residual intramedullary neoplasm with enhancement identified. There was no metastasis to the lumbosacral region identified. Degenerative changes in the lower lumbar spine were noted.

**Figure 5:**
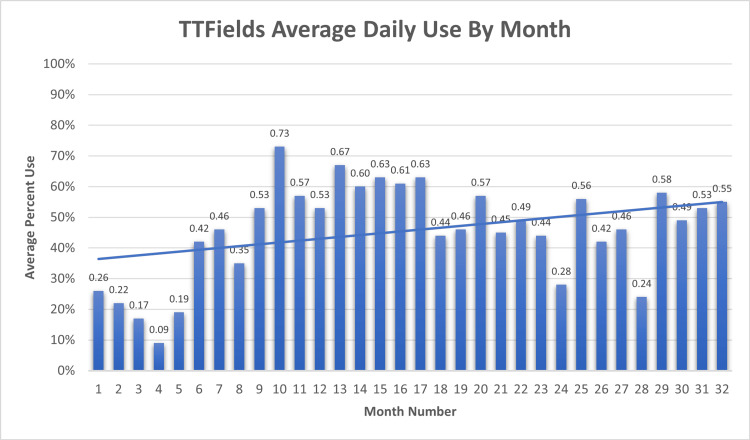
Novocure Optune TTFields device usage The 32-month period began five months postoperatively and continued through approximately mid-2023. Total average daily use: 46%

The patient began TTFields treatment with the Novocure Optune device approximately five months postoperatively. Her daily average TTFields treatment time fluctuated, as depicted in Figure 5 below.

**Figure 6:**
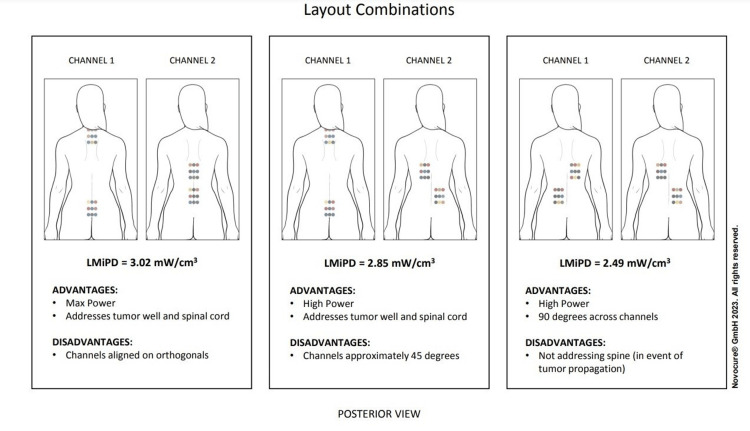
The TTFields array layout combinations indicate the local minimum power density (LMiPD). Language for figure legends: Reused with permission from © 2023 Novocure GmbH, all rights reserved.

In the first three months, her TTFields use was limited due to skin irritation on her back from array placement. This skin irritation was treated medically with skin-protectant wipes and clobetasol 0.05% cream. Overall, her TTFields usage was less than the minimum recommended 18 hours daily [3,4] (75%), with her total average use being 46% over the 32-month period reported. Figure 6 indicates the TTFields array layout combinations.

One month following concurrent chemotherapy and radiation therapy, the patient began adjuvant chemotherapy with temozolomide. The temozolomide dose was adjusted between 150 mg/m^2^ and 75 mg/m^2 ^by the medical oncologist as the patient experienced side effects of nausea and vomiting. She continues maintenance chemotherapy with temozolomide under close monitoring by medical oncology. She continues maintenance TTFields treatment to her spine.

At 32 months post-completion of radiation therapy, she reports continued significant improvement in ambulation. She reports no new neurological symptoms. She continues to have paresthesia involving her right lower extremity, which is managed with gabapentin. She denies bowel or bladder deficits. Early in her treatment, the patient had significant painful neuropathy, mostly involving the right lower extremity, as well as chronic pain and muscle spasms in both legs, which were managed medically. She received physical therapy and continues to be followed regularly by a pain management specialist.

**Figure 7:**
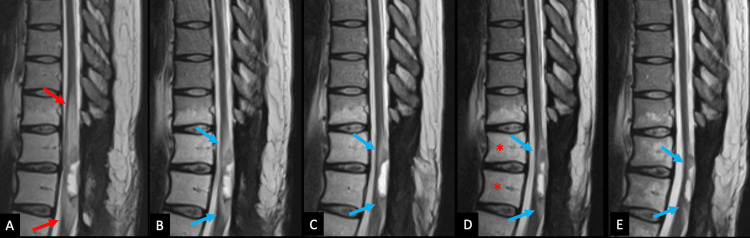
Post-concurrent radiation and chemotherapy MR imaging Serial surveillance MR imaging of the thoracic spine. The patient completed radiation therapy to T10 through L1 levels. At the one-month follow-up exam, the sagittal (A) T2 weighted image (T2WI) demonstrates an increase in vasogenic edema surrounding the tumor (red arrows). This gradually decreases in extent over follow-up exams (blue arrows) at four months (B), seven months (C), and 10 months (D). It remains stable over subsequent exams, with the most recent follow-up at 28 months (E). There is an expected fat-signal change in the bone marrow from T10 to L1 related to radiation therapy (red asterisks, D).

**Figure 8:**
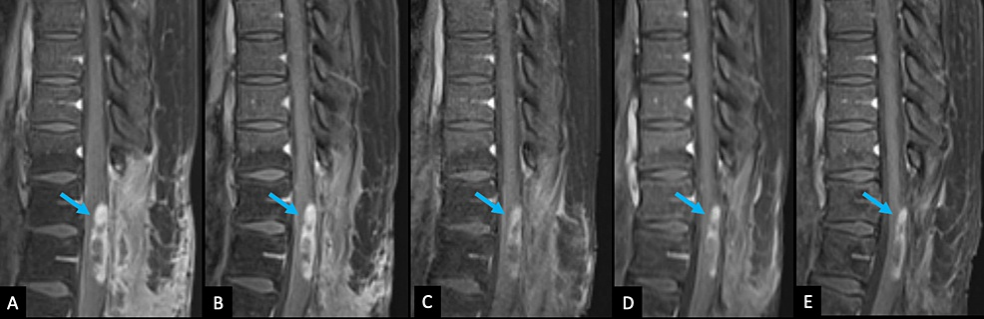
Post-concurrent radiation and chemotherapy MR imaging Serial surveillance MR imaging of the thoracic spine. On the fat-suppressed, post-contrast sagittal T1 weighted image (T1WI), there is progressively decreasing contrast enhancement (blue arrows) associated with the residual tumor at one month (A), four months (B), seven months (C), and 10 months (D). It remains stable over subsequent exams, with the most recent follow-up at 28 months (E).

Some weeks following the commencement of TTFields treatment, an MRI of her lumbar spine showed the partially visualized known intramedullary spinal cord mass with no abnormal enhancement in the lumbar spine. Serial surveillance MRIs (Figures 7, 8) showed a progressive decrease in the size of the intramedullary heterogeneously enhancing mass at the T10 through T12 levels from approximately 1.1 x 0.7 x 4.1 cm to 0.7 x 0.7 x 3.7 cm.

There was also a decrease in edema in the distal thoracic to lumbar regions. There was no evidence of metastasis in the lumbar spine region. The patient’s most recent MRI of the thoracic spine at 28 months following radiation shows stable disease.

## Discussion

Gliomas account for approximately 25% of all primary brain tumors and more than 80% of all malignant CNS tumors [5]. Glioblastoma is the most common malignant primary brain tumor. It is the most aggressive type of glioma, representing 57% of this group [5]. Glioblastoma multiforme progresses rapidly, with only one in four patients alive at two years and only 5% to 10% of patients alive at five years from diagnosis [4]. While GBM is most commonly intracranial, primary spinal GBM in WHO classification IV is relatively rare, accounting for only 1%-3% of primary spinal cord tumors [6,7].

The treatment for newly diagnosed supratentorial GBM usually includes the safest maximal debulking surgery, followed by treatment with radiation and chemotherapy per NCCN guidelines [2]. In recent years, alternating electric field therapy has been approved by the FDA and is now indicated in the adjuvant management of supratentorial GBM [2]. Per the NCCN recommendations, alternating electrical field therapy is indicated as an adjuvant treatment in conjunction with temozolomide chemotherapy for newly diagnosed supratentorial glioblastoma multiforme (GBM) following maximal debulking surgery and radiation [2]. Tumor-treating fields are also indicated as an alternative to standard medical therapy in recurrent GBM when surgery and radiation options have been exhausted [2].

On the other hand, likely owing to the rarity of primary spinal GBM and the impracticality of performing strong clinical trials for its treatment, treatment protocol choices for this disease entity remain controversial but typically include surgery, radiation therapy, and chemotherapy [7]. Hence, treatment protocols for spinal GBM generally mirror those used for intracranial GBM [6].

In a multicenter, open-label, randomized clinical phase 3 trial, Stupp et al. found that TTFields treatment combined with maintenance temozolomide resulted in statistically significant prolonged disease-free survival and overall survival vs. temozolomide alone in the treatment of intracranial GBM [4]. This trial enrolled 695 patients with intracranial GBM who had undergone resection or biopsy and had completed concomitant radiochemotherapy. These patients were randomized 2:1 to receive maintenance temozolomide plus TTFields or maintenance temozolomide alone. According to Stupp et al., the median progression-free survival in the TTFields-temozolomide group was 6.7 months vs. 4.0 months in the temozolomide-alone group [4]. Furthermore, median overall survival was 20.9 months in the combination treatment arm vs. 16.0 months in the temozolomide-alone group.

In the management of spinal GBM, Shen et al. report a case of primary spinal GBM in a 15-year-old female found to have intramedullary GBM localized between C4 and C7 [7]. Per Shen’s report, this patient had a partial tumor resection followed by focal adjuvant radiotherapy concomitantly with oral chemotherapy. Unfortunately, with severe neurologic deficits, the patient expired 13 months after diagnosis.

In a case series at one institution, Nagarajan and Ravichandar report three cases of spinal GBM [8]. One of the patients, a 32-year-old male, was found to have a GBM lesion at the C1-C4 level but had no evidence of a neurological deficit. This patient underwent radical surgery followed by adjuvant radiotherapy with concurrent temozolomide, followed by adjuvant temozolomide. Nagarajan and Ravichandar reported that this patient had the longest disease-free survival of 96 months, to the best of their knowledge at the time. Their second case was a 27-year-old male with spinal GBM at C2-C3 level who underwent partial excision followed by radiotherapy along with concurrent temozolomide. Unfortunately, this patient succumbed to the progressive illness after the first cycle of adjuvant temozolomide. The third patient in this series was a 13-year-old female found to have cervicothoracic “C7-D7” [8] GBM who underwent excision of the lesion, followed by postoperative radiotherapy with concurrent temozolomide but no adjuvant temozolomide due to parental refusal. This patient had a 16-month progression-free survival and was still being followed, per Nagarajan and Ravichandar.

In the case of our patient, we opted to apply Stupp et al.’s treatment [4]. In summary, following surgery, our patient received concurrent radiation therapy and temozolomide. Given the statistically significant prolonged survival reported with TTFields treatment in intracranial GBM by Stupp et al. [4], we opted to employ the compassionate use of Novocure TTFields as an adjuvant treatment concurrently with cycles of adjuvant temozolomide as well. Our patient is continuing with this maintenance regimen. She has recovered significantly from her lower extremity weakness and is able to ambulate independently despite some residual numbness in the right lower extremity. 

Tumor-treating fields are a non-invasive therapy for solid tumors that uses low-intensity, intermediate-frequency alternating electric fields to physically disrupt processes vital for the division of cancer cells. The frequency range of TTFields is between 100 kHz and 500 kHz, a range that does not stimulate or heat tissue but can enter cells for biological effect [9]. Once in the cell, TTFields exert physical forces on polar cellular components, such as the cytoskeletal elements, cell membranes, and DNA molecules [1,10]. These physical forces also affect the orientation and movement of the mitotic spindle during the mitotic process, causing the spindle to align in the direction of the electric field, disrupting its normal function, and leading to cellular stress and immunogenic forms of cancer cell death [11-13]. 

Tumor-treating fields also alter the cell's membrane potential, affecting ion channels and transporters. This alteration in the membrane potential can lead to changes in cell signaling pathways and gene expression, which can contribute to the anti-cancer effects of TTFields [14,15]. A potentially anti-metastatic effect for TTFields has also been observed in cancer cell lines, where TTFields have been shown to interfere with cancer cell motility by impairing the organization and dynamics of the microtubule network [13].

Tumor-treating fields have been shown to alter the expression of genes involved in cell division, cell cycle control, and DNA damage repair [15,16]. These changes can contribute to the inhibition of tumor growth and the induction of cell death. Tumor-treating fields can also induce autophagy, a cellular process that involves the degradation of damaged or dysfunctional cellular components. Autophagy can lead to the death of cancer cells and the reduction of tumor growth [17]. Thus, the physical effects of TTFields on cells and tissues involve a combination of multiple physical and biological processes. These multiple anticancer cell mechanisms for TTFields are the subject of ongoing studies and underscore many potential synergies with current cancer therapies.

At the time of this publication, our patient had a 32-month progression-free survival. Her prolonged survival may correlate to the aforementioned multiple treatment modalities employed, the thoracic location of her disease, which, per Shen et al., proved to have a more favorable prognosis compared to cervical tumors [7], as well as the multidisciplinary approach to her management. A question to consider is whether the stability of her disease is attributable to her prolonged maintenance of temozolomide or her maintenance TTFields, versus the combination of both treatment modalities. Of note, to the best of our knowledge, our case may only be the first one where TTFields is being employed in the treatment of spinal GBM and the second one where TTFields is employed in the treatment of a spinal cord tumor. The first similar use of TTFields in spinal astrocytoma was reported by De Los Santos et al. in a poster presented at the Society for Neuro-Oncology (SNO) 2020 Virtual Meeting [18].

There remains a need for standardized treatment for spinal GBM. Treatment modalities applied to supratentorial GBM should be further studied for their applicability to spinal GBM. Tumor-treating fields, which have been shown to prolong disease-free survival and overall survival in supratentorial GBM, could also be beneficial in spinal GBM. From our use of TTFields for spinal GBM in our patient, TTFields appears to be a safe adjunct treatment modality for spinal GBM. In agreement with Timmons et al. [6], due to the rarity of spinal GBM, multiple randomized controlled trials may not be feasible. Therefore, case reports of spinal GBM, regardless of outcome, should continue to be published with appropriate detail, including a timeline of treatment.

## Conclusions

While supratentorial GBM is the most common CNS malignancy, spinal GBM is rare. Regardless of location, GBM has a very poor prognosis, even with treatment. Though guidelines are available for the management of supratentorial GBM, there is still a lack of consensus on treatment guidelines for spinal GBM. Here we presented the case of a young female diagnosed with primary spinal GBM in the thoracic region, whose treatment mirrored that of supratentorial GBM. We further employed maintenance temozolomide with concurrent alternating electric field therapy, with a favorable progression-free survival outcome. From our use of TTFields treatment for spinal GBM in our patient, TTFields appear to be a safe adjunct treatment modality for spinal GBM. However, further studies of TTFields treatment in spinal GBM are needed. Moreover, more studies are needed aimed at finding improved treatments for primary spinal GBM.
